# 1α,25-Dihydroxyvitamin D_3 _enhances cerebral clearance of human amyloid-β peptide(1-40) from mouse brain across the blood-brain barrier

**DOI:** 10.1186/2045-8118-8-20

**Published:** 2011-07-08

**Authors:** Shingo Ito, Sumio Ohtsuki, Yasuko Nezu, Yusuke Koitabashi, Sho Murata, Tetsuya Terasaki

**Affiliations:** 1 Division of Membrane Transport and Drug Targeting, Graduate School of Pharmaceutical Sciences, Tohoku University, Aoba, Aramaki, Aoba-ku, Sendai 980-8578, Japan; 2 SORST of the Japan Science and Technology Agency, Japan

## Abstract

**Background:**

Cerebrovascular dysfunction has been considered to cause impairment of cerebral amyloid-β peptide (Aβ) clearance across the blood-brain barrier (BBB). Further, low levels of vitamin D are associated with increased risk of Alzheimer's disease, as well as vascular dysfunction. The purpose of the present study was to investigate the effect of 1α,25-dihydroxyvitamin D_3 _(1,25(OH)_2_D3), an active form of vitamin D, on cerebral Aβ clearance from mouse brain.

**Methods:**

The elimination of [^125^I]hAβ(1-40) from mouse brain was examined by using the Brain Efflux Index method to determine the remaining amount of [^125^I]hAβ(1-40) radioactivity after injection into the cerebral cortex. [^125^I]hAβ(1-40) internalization was analyzed using conditionally immortalized mouse brain capillary endothelial cells (TM-BBB4).

**Results:**

Twenty-four hours after intraperitoneal injection of 1,25(OH)_2_D3 (1 μg/mouse), [^125^I]hAβ(1-40) elimination from mouse brain was increased 1.3-fold, and the level of endogenous Aβ(1-40) in mouse brain was reduced. These effects were observed at 24 h after i.p. injection of 1,25(OH)_2_D3, while no significant effect was observed at 48 or 72 h. Vitamin D receptor (VDR) mRNA was detected in mouse brain capillaries, suggesting that 1,25(OH)_2_D3 has a VDR-mediated genomic action. Furthermore, forskolin, which activates mitogen-activated protein kinase kinase (MEK), enhanced [^125^I]hAβ(1-40) elimination from mouse brain. Forskolin also enhanced [^125^I]hAβ(1-40) internalization in TM-BBB4 cells, and this enhancement was inhibited by a MEK inhibitor, suggesting involvement of non-genomic action.

**Conclusions:**

The active form of vitamin D, 1,25(OH)_2_D3, appears to enhance brain-to-blood Aβ(1-40) efflux transport at the BBB through both genomic and non-genomic actions. Compounds activating these pathways may be candidate agents for modulating Aβ(1-40) elimination at the BBB.

## Background

An abnormally elevated level of amyloid-β peptide (Aβ) in the brain is one of the prominent features of Alzheimer's disease (AD) [1]. Aβ is normally produced by neurons and cleared through degradation by proteinases within the brain [2-4], as well as through elimination from the brain to the circulating blood via an efflux transport system at the blood-brain barrier (BBB) [5,6]. It has been proposed that impairment of cerebral Aβ clearance leads to abnormally elevated brain Aβ levels in late-onset AD which accounts for more than 90% of all cases of AD. On the other hand, somatostatin was reported to promote the degradation of Aβ in the brain [7]. Anti-Aβ antibodies such as m266 in circulating blood were also proposed to enhance Aβ efflux transport from brain to blood [8], though we subsequently found that anti-Aβ antibodies did not promote Aβ elimination from brain to the blood across the BBB [9]. Identification of factors that promote cerebral Aβ clearance would be helpful in developing new therapeutic approaches and agents for treatment and prevention of AD.

Epidemiological studies have suggested that a low level of serum 25-hydroxyvitamin D_3 _(25(OH)D3), the major circulating form of vitamin D, is associated with cognitive impairment in elderly persons [10-12] and is a risk factor for AD [13,14]. Furthermore, it was reported that single nucleotide polymorphisms in the vitamin D receptor (VDR) gene increase the risk of impairment of cognitive function and developing AD [15,16], suggesting a relation between serum vitamin D levels and risk of AD. It was also reported that low levels of serum 25(OH)D3 are associated with increased risk for cardiovascular diseases, hypertension and diabetes mellitus [17-20], which are associated with vascular dysfunction. Since clinical studies have provided evidence that vascular dysfunction plays an important role in early progression of AD [21-23], we hypothesized that serum vitamin D affects the function of brain capillaries, including the brain-to-blood efflux transport of Aβ.

The purpose of the present study was to investigate the effect of 1α,25-dihydroxyvitamin D_3 _(1,25(OH)_2_D3), the physiologically active form of vitamin D, on the elimination of human Aβ(1-40) (hAβ(1-40)) from mouse brain across the BBB using the Brain Efflux Index (BEI) method. We also examined the internalization of [^125^I]hAβ(1-40) in conditionally immortalized mouse brain capillary endothelial cells (TM-BBB4), as an *in vitro *BBB model [24].

## Methods

### Animals

Male C57BL/6 mice (8-10 weeks old) were purchased from Japan SLC (Hamamatsu, Japan). All experiments were approved by the Animal Care Committee of the Graduate School of Pharmaceutical Sciences, Tohoku University.

### Reagents

Monoiodinated, non-oxidized and lyophilized [^125^I]hAβ(1-40) labeled from hAβ(1-40) (Biosource, CA, USA) was from PerkinElmer Life Sciences (Boston, MA, USA) (2,200 Ci/mmol). [^3^H]Dextran (100 mCi/mg) was obtained from American Radiolabeled Chemicals, Inc. (St. Louis, MO, USA). 1,25(OH)_2_D3 was obtained from Biomol Research Laboratories (Plymouth Meeting, PA, USA). Forskolin and xylazine hydrochloride were purchased from Sigma (St Louis, MO, USA). U0126 was obtained from Calbiochem (Merck, Darmstadt, Germany). Ketaral 50 (ketamine hydrochloride) was purchased from Sankyo Co. (Tokyo, Japan). All other chemicals were analytical grade commercial products.

### In vivo BEI study

The mice were injected i.p. with 100 μL of 1,25(OH)_2_D3 at a dose of 0.2, 1 or 5 μg dissolved in H_2_O containing 5% ethanol, or forskolin at a dose of 2.3 or 23 μg dissolved in 100 μL of H_2_O containing 25% dimethyl sulfoxide. Control mice were injected i.p. with 100 μL of each vehicle solution. After an indicated time (24, 48, or 72 h), the *in vivo *brain elimination experiments were performed using the intracerebral microinjection technique [25]. Briefly, mice were anesthetized with an intramuscular injection of xylazine (1.22 mg/kg) and ketamine (125 mg/kg), and placed in a stereotaxic frame (SRS-6; Narishige, Tokyo, Japan) that determines the coordinates of the mouse brain coinciding with the secondary somatosensory cortex 2 (S2) region. A small hole was made 3.8 mm lateral to the bregma, and a fine injection needle fitted to a 5.0 μl microsyringe (Hamilton, Reno, NE, USA) was advanced to a depth of 2.5 mm. The solution of [^125^I]hAβ(1-40) was freshly prepared from lyophilized [^125^I]hAβ(1-40) immediately before each experiment. Lyophilized [^125^I]hAβ(1-40) was solubilized in H_2_O and then added to an equal volume of 2-fold concentration of extracellular fluid (ECF) buffer (244 mM NaCl, 50 mM NaHCO_3_, 6 mM KCl, 2.8 mM CaCl_2_, 2.4 mM MgSO_4_, 0.8 mM K_2_HPO_4_, 20 mM D-glucose, and 20 mM HEPES, pH 7.4). Gel filtration analysis confirmed that a single peak of monomer [^125^I]hAβ(1-40) was detected, and 98.3% of the radioactivity was recovered, suggesting that release of free [^125^I] was negligible [26]. The applied solution (0.3 μl) containing [^125^I]hAβ(1-40) and [^3^H]dextran in an ECF buffer was administered into the S2 region over a period of 30 sec. After microinjection, the microsyringe was left in place for 4 min to minimize any backflow. At 5, 30 or 60 min after microinjection, ipsilateral (left) and contralateral (right) cerebrum and cerebellum were excised and dissolved in 2.0 ml 2 M NaOH at 60°C for 1 h. The ^125^I and ^3^H radioactivities of the samples were measured in a γ-counter (ART300, Aloka, Tokyo, Japan) for 3 min and a liquid scintillation counter (TRI-CARB2050CA, Packard Instruments, Meriden, CT, USA) for 5 min, respectively. The BEI was defined by Equation (1), and the percentage of substrate remaining in the ipsilateral cerebrum (100-BEI) was determined using Equation (2):

The apparent elimination rate constant (k_el_) was determined from the slope given by fitting a semilogarithmic plot of (100-BEI) versus time, using the nonlinear least-squares regression analysis program MULTI [27].

### Quantification of endogenous soluble mouse Aβ(1-40) by ELISA

Endogenous soluble Aβ(1-40) in mouse brain extracts was quantified according to the previously-reported method [28]. Twenty-four h after 1,25(OH)_2_D3 (1 μg/mouse) or vehicle injection i.p., mice were sacrificed and the brains were rapidly isolated. The cerebellum and olfactory bulbs were discarded, and the remaining brains were snap-frozen in liquid nitrogen. To extract soluble endogenous full-length mouse Aβ(1-40), the frozen brains were homogenized in 10 volumes of ice-cold Tris buffer (50 mM Tris-HCl, 250 mM sucrose and protease inhibitor cocktail (Sigma, St. Louis, MO, USA)). Samples were mixed in diethylamine (DEA) to yield 0.4% concentration and centrifuged at 100,000 × *g *for 45 min at 4°C. The resultant supernatant was neutralized in 1/10 volume of 0.5 M Tris-HCl (pH 6.8) and used for analysis. Endogenous full-length mouse Aβ(1-40) levels were determined using an ELISA (IBL, Gunma, Japan) according to the manufacturer's protocol.

### TM-BBB4 cell culture

The TM-BBB4 cell line, established from transgenic mice harboring the temperature-sensitive SV40 large T-antigen gene [24], was used in this study. TM-BBB4 cells were cultured at 33°C in Dulbecco's modified Eagle's medium (DMEM; Nissui Pharmaceutical Co., Tokyo, Japan), supplemented with 20 mM NaHCO_3_, 2 mM L-glutamine, 15 ng/ml endothelial cell growth factor, 100 U/ml benzyl penicillin, 100 mg/ml streptomycin sulfate, and 10% fetal bovine serum (Moregate, Bulimba, Australia) in an atmosphere of 95% air and 5% CO_2_.

### RT-PCR analysis

Isolated mouse brain capillaries were prepared as described previously [29]. Total RNA was prepared from isolated mouse brain capillaries, TM-BBB4 cells and mouse brain using TRIzol reagent (Life Technologies, Grand Island, NY, USA) and RNeasy plus kit (Qiagen, Tokyo, Japan) according to the manufacturer's protocols. Single-stranded cDNA was prepared from 1 μg of total RNA by means of reverse transcription (DNA Ligation Kit, Takara, Osaka, Japan) using oligo dT primer. The PCR was performed with GeneAmp (PCR system 9700, Perkin-Elmer, Norwalk, CT, USA) using specific primers through 1 cycle of 94°C for 2 min, and 35 cycles of 94 °C for 30 s, 55°C for 30 s and 72°C for 1 min and finally 72°C for a further 5 min. The sequences of the primers were as follows: sense primer 5'-CGA TGC CCA CCA CAA GAC CTAC-3' and antisense primer 5'-CAG CAT GGA GAG CGG AGA CAG-3' for vitamin D receptor (GenBank Accession Number, NM_009504), sense primer 5'-TTT GAG ACC TTC AAC ACCC C-3' and antisense primer 5'-ATA GCT CTT CTC CAG GGA GG-3' for β-actin (GenBank Accession Number, NM_031144). The RT-PCR of each sample RNA in the absence of reverse transcriptase was used as a negative control. The RT-PCR products were separated by electrophoresis on an agarose gel in the presence of ethidium bromide (0.6 μg/mL) and visualized using an imager (EPIPRO 7000; Aisin, Aichi, Japan).

### In vitro internalization study in TM-BBB4 cells

TM-BBB4 cells were seeded on 24-well plates (Becton Dickinson, Bedford, MA, USA) at a density of 1.0 × 10^5 ^cells/2 cm^2^/well and cultured for 24 h. The cells were treated with the indicated concentrations of forskolin and U0126 or vehicle for 24 h, and then internalization of [^125^I]hAβ(1-40) into TM-BBB4 cells was examined as previously described [26]. Briefly, the extent of TM-BBB4 cell-[^125^I]hAβ(1-40) (0.15 μCi/200 μl in ECF buffer) binding was measured for 3 min at 37°C. After the indicated times, binding was terminated by removing the solution, and the cells were washed three times with 1 ml ice-cold ECF buffer. The cells were then incubated with 1 ml ice-cold acetate-barbital buffer (28 mM CH_3_COONa, 120 mM NaCl, 20 mM barbital sodium (pH 3.0) and 360 mOsm/kg) for 20 min at 4°C to remove [^125^I]hAβ(1-40) bound to the cell surface. After incubation, the buffer was then recovered (acid-soluble fraction) and the cells were subsequently washed three times with ice-cold acetate-barbital buffer.

Acid-resistant binding represents the amount of internalized [^125^I]hAβ(1-40) in the TM-BBB4 cells. The cells were solubilized with 200 μl of 5 M NaOH overnight and neutralized with 200 μl of 5 M HCl. The ^125^I radioactivity was measured using a γ-counter (ART310, Aloka, Tokyo, Japan). The protein content of the cultured cells was measured using a DC protein assay kit (Bio-Rad, Hercules, CA, USA) with bovine serum albumin as a standard.

### Data analysis

Unless otherwise indicated, all data represent the mean ± SEM. Unpaired two-tailed Student's *t-*tests were used to determine the significance of differences between the means of two groups. One-way analysis of variance followed by Dunnett's test was used to assess the statistical significance of differences among the means of more than two groups.

## Results

### Effect of 1,25(OH)_2_D3 on the brain-to-blood efflux transport rate of [^125^I]hAβ(1-40) at the BBB

**Figure 1:**
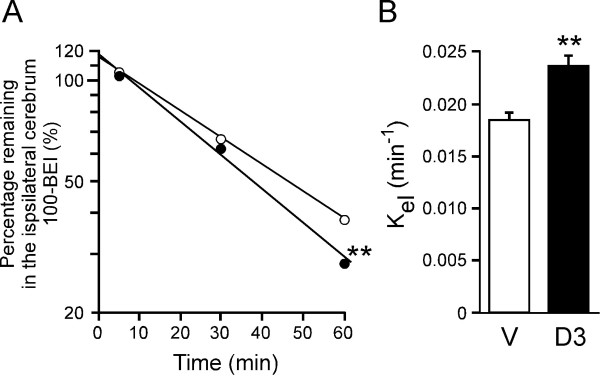
**Effect of 1,25(OH)_2_D3 on the elimination rate of [^125^I]hAβ(1-40) from mouse brain**. Mice received i.p. injection of 1,25(OH)_2_D3 (1 μg dissolved in 100 μL of H_2_O containing 5% ethanol, solid circles) or vehicle (100 μL of H_2_O containing 5% ethanol, open circles). After 24 h, a mixture of [^125^I]hAβ(1-40) (0.012 μCi) and [^3^H]dextran (0.12 μCi) dissolved in 0.30 μL of ECF buffer was injected into the S2 region of the brain. The solid line was obtained with the non-linear least-squares regression analysis program, MULTI. Each point represents the mean ± SEM (n = 3 - 5). (B) The elimination rate constant of [^125^I]hAβ(1-40) from mouse brain after treatment with 1,25(OH)_2_D3 (D3) or vehicle (V). Each bar represents the mean ± SD (n = 3 - 5). **p < 0.01, significantly different from vehicle-treated group.

To examine the effect of 1,25(OH)_2_D3 treatment on brain-to-blood [^125^I]hAβ(1-40) efflux transport across the BBB, the time profile of the percentage of [^125^I]hAβ(1-40) remaining in the ipsilateral cerebrum was examined at 24 h after i.p. administration of a single dose of 1,25(OH)_2_D3 (1 μg/mouse) (Figure 1). [^125^I]hAβ(1-40) was eliminated from the brain in a time-dependent manner after the administration of either 1,25(OH)_2_D3 or vehicle (Figure 1A). The percentage of [^125^I]hAβ(1-40) remaining in 1,25(OH)_2_D3-treated mouse brain was significantly lower than that in the vehicle-treatment group at 60 min. The apparent elimination rate constant across the BBB of [^125^]hAβ(1-40) determined from the slope of the (100-BEI (%)) time profile was 2.38 × 10^-2 ^± 0.09 × 10^-2 ^min^-1 ^(mean ± SD) in the 1,25(OH)_2_D3-treated group, which was significantly greater (1.29-fold) than that of the vehicle-treated group (1.85 × 10^-2 ^± 0.07 × 10^-2 ^min^-1^, mean ± SD) (Figure 1B). The initial percentage of [^125^I]hAβ(1-40) remaining in the mouse brain treated with 1,25(OH)_2_D3 was 118% (Figure 1A), which is not significantly different from that of the vehicle-treated group (114%), indicating that the distribution volume of [^125^I]hAβ(1-40) in the brain was not affected by 1,25(OH)_2_D3 treatment. This suggests that 1,25(OH)_2_D3-treatment did not affect hAβ(1-40) binding to brain parenchymal cells in mouse brain.

### Dose and time dependence of 1,25(OH)_2_D3 action on [^125^I]hAβ(1-40) elimination from mouse brain

**Figure 2:**
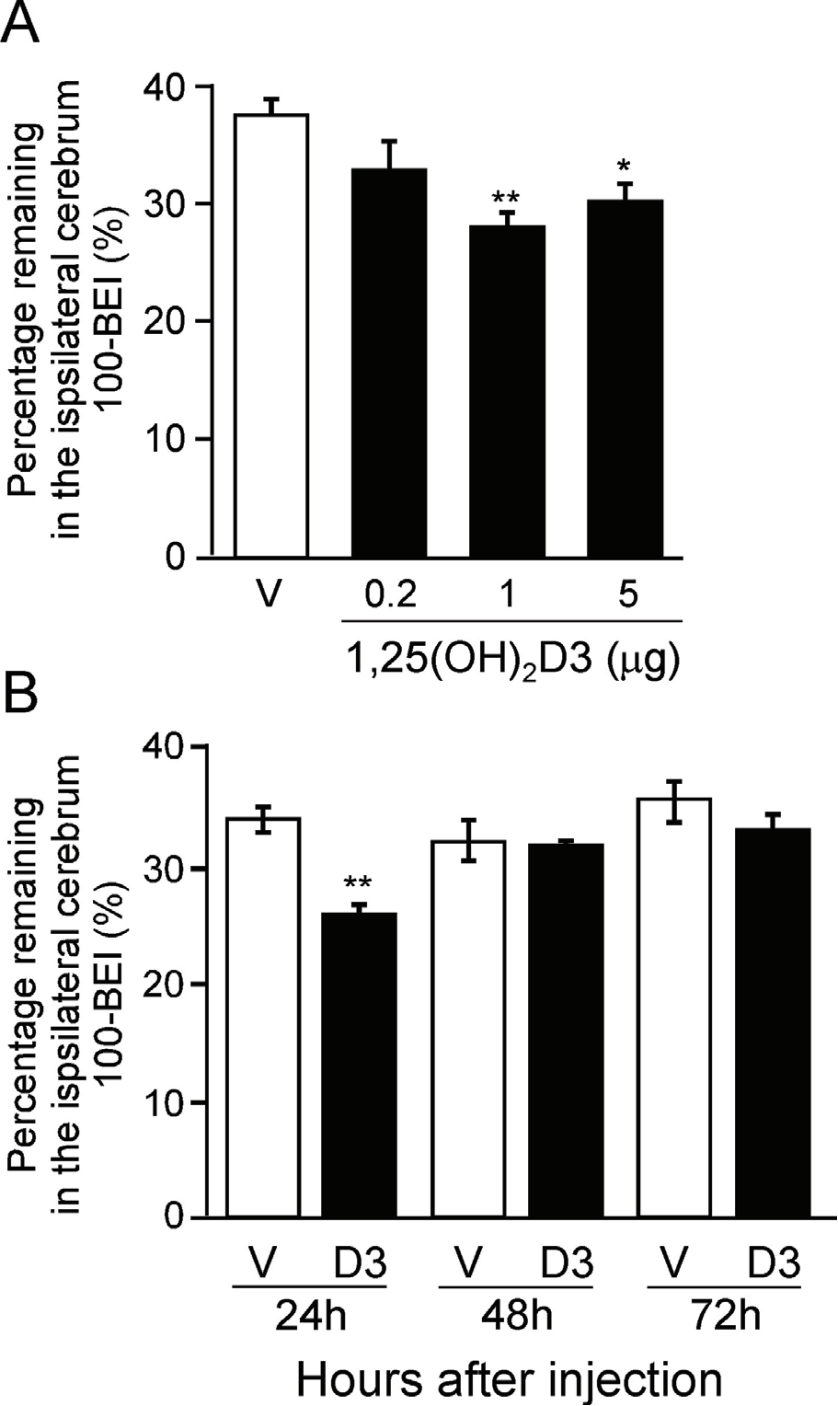
**Effect of 1,25(OH)_2_D3 on amount of [^125^I]hAβ(1-40) remaining in the ipsilateral cerebrum**. (A) Dose-dependence of the effect of 1,25(OH)_2_D3 on [^125^I]hAβ(1-40) elimination from mouse brain. Mice received i.p. injection of 1,25(OH)_2_D3 (0.2, 1 or 5 μg dissolved in 100 μL of H_2_O containing 5% ethanol) or vehicle (V, 100 μL of H_2_O containing 5% ethanol). After 24 h, a mixture of [^125^I]hAβ(1-40) (0.012 μCi) and [^3^H]dextran (0.12 μCi) dissolved in 0.30 μL of ECF buffer was injected into the S2 region of the brain. Data, obtained at 60 min after intracerebral microinjection, are presented as the mean ± SEM (n = 5). (B) Time-course of the effect of i.p. administration of 1,25(OH)_2_D3 on [^125^I]hAβ(1-40) elimination from mouse brain. Mice received i.p. injection of 1,25(OH)_2_D3 (D3, 1 μg dissolved in 100 μL of H_2_O containing 5% ethanol) or vehicle (V, 100 μL of H_2_O containing 5% ethanol). After 24, 48 and 72 h, a mixture of [^125^I]hAβ(1-40) (0.012 μCi) and [^3^H]dextran (0.12 μCi) dissolved in 0.30 μL of ECF buffer was injected into the S2 region of the brain. Data, obtained at 60 min after intracerebral microinjection, are presented as the mean ± SEM (n = 4 - 5). *p < 0.05, **p < 0.01, significantly different from vehicle-treated group.

Figure 2A shows the dose-dependence of 1,25(OH)_2_D3's effect on the percentage of [^125^I]hAβ(1-40) remaining in the ipsilateral cerebrum at 60 min after microinjection; this time point was chosen because the percentage of [^125^I]hAβ(1-40) remaining decreased time-dependently up to 60 min (Figure 1A, open circles). Twenty-four h after single i.p. administration of 1,25(OH)_2_D3 (0.2, 1 and 5 μg/mouse), the percentage of [^125^]hAβ(1-40) remaining was decreased by 12.9%, 25.4% and 19.5%, respectively, compared with the vehicle-treated control (Figure 2A).

The effect of time after 1,25(OH)_2_D3 administration was examined at the amount of 1 μg/mouse, which exhibited the greatest effect in Figure 2A. As shown in Figure 2B, a significant reduction of the percentage of [^125^]hAβ(1-40) remaining was observed at 24 h after i.p. administration of a single dose of 1,25(OH)_2_D3, compared with the vehicle-treated control, while no significant reduction was detected at 48 or 72 h after the i.p. administration. No significant decrease was observed in body weight or residual amount of [^3^H]dextran, which is an impermeable marker, in mouse brain up to 72 h after the administration (data not shown), suggesting that a single dose of 1,25(OH)_2_D3 did not cause significant toxicity or disruption of BBB integrity.

#### Effect of 1,25(OH)_2_D3 on the level of endogenous Aβ(1-40) in mouse brain

**Figure 3:**
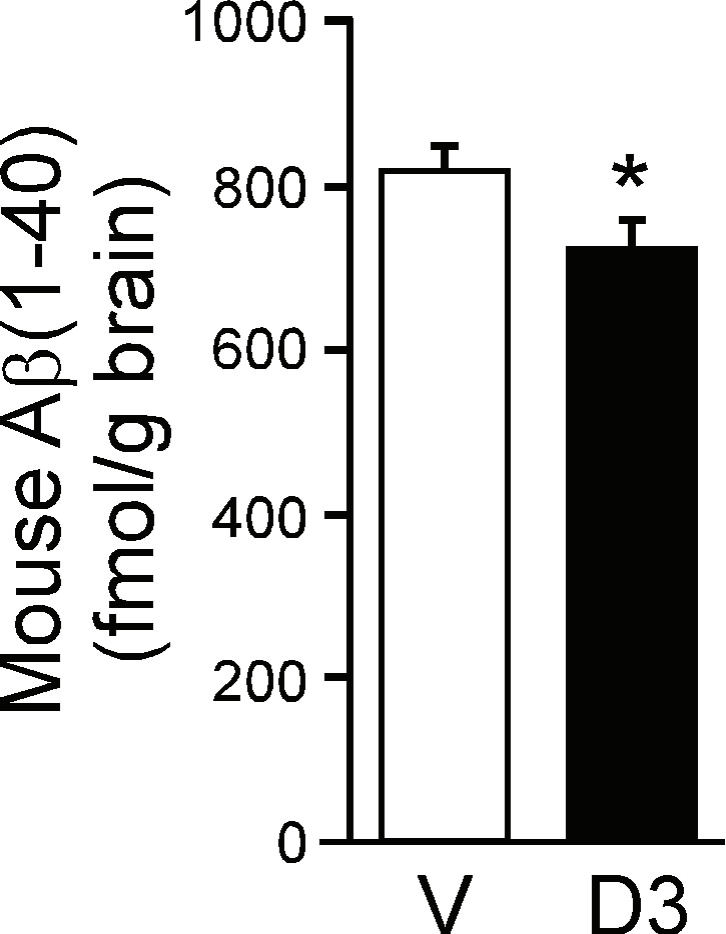
**Effect of 1,25(OH)_2_D3 on the level of endogenous Aβ(1-40) in mouse brain**. Mice received i.p. injection of 1,25(OH)_2_D3 (D3, 1 μg dissolved in 100 μL of H_2_O containing 5% ethanol) or vehicle (V, 100 μL of H_2_O containing 5% ethanol). After 24 h, brain levels of DEA-extracted mouse Aβ(1-40) were measured by ELISA. Each bar represents the mean ± SEM (n = 8). *p < 0.05, significantly different from vehicle-treated group.

The effect of 1,25(OH)_2_D3 on soluble endogenous Aβ(1-40) level in mouse brain was examined by ELISA. Endogenous Aβ(1-40) extracted with 0.4% DEA was significantly decreased at 24 h after i.p. administration of a single dose of 1,25(OH)_2_D3 compared with the vehicle-treated group; the values were 723 ± 34 and 821 ± 30 fmol/g brain, respectively (p < 0.05, Figure 3).

### mRNA expression of VDR in mouse brain, brain capillaries and TM-BBB4 cells

**Figure 4:**
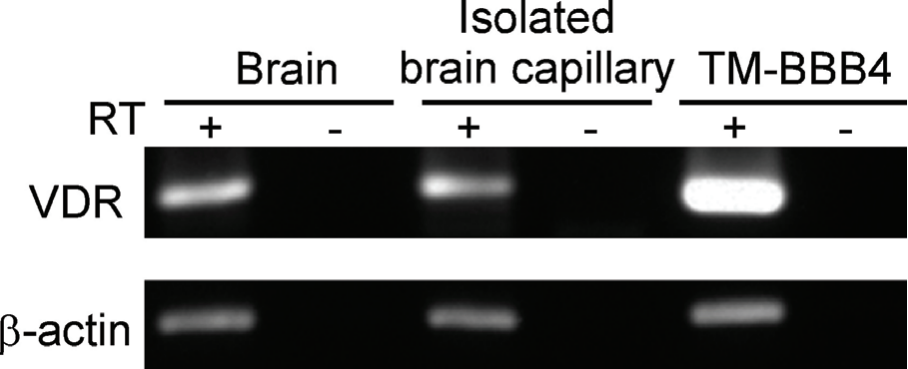
**Expression of mRNAs for VDR and β-actin in mouse brain, isolated brain capillaries and TM-BBB4 cells**. RT-PCR analysis was performed with specific primer sets as described in Materials and Methods. Reactions were performed using total RNA with (+) or without (-) reverse transcriptase (RT).

1,25(OH)_2_D3 exerts its biological effects by transcriptional activation of target genes via binding to the VDR. RT-PCR analysis was performed to determine VDR expression in mouse brain, mouse isolated brain capillaries and TM-BBB4 cells [24]. A band of the size predicted for VDR was obtained in each case (Figure 4), suggesting that VDR is expressed in mouse brain capillary endothelial cells.

### Effect of forskolin on [^125^I]hAβ(1-40) elimination from mouse brain

**Figure 5:**
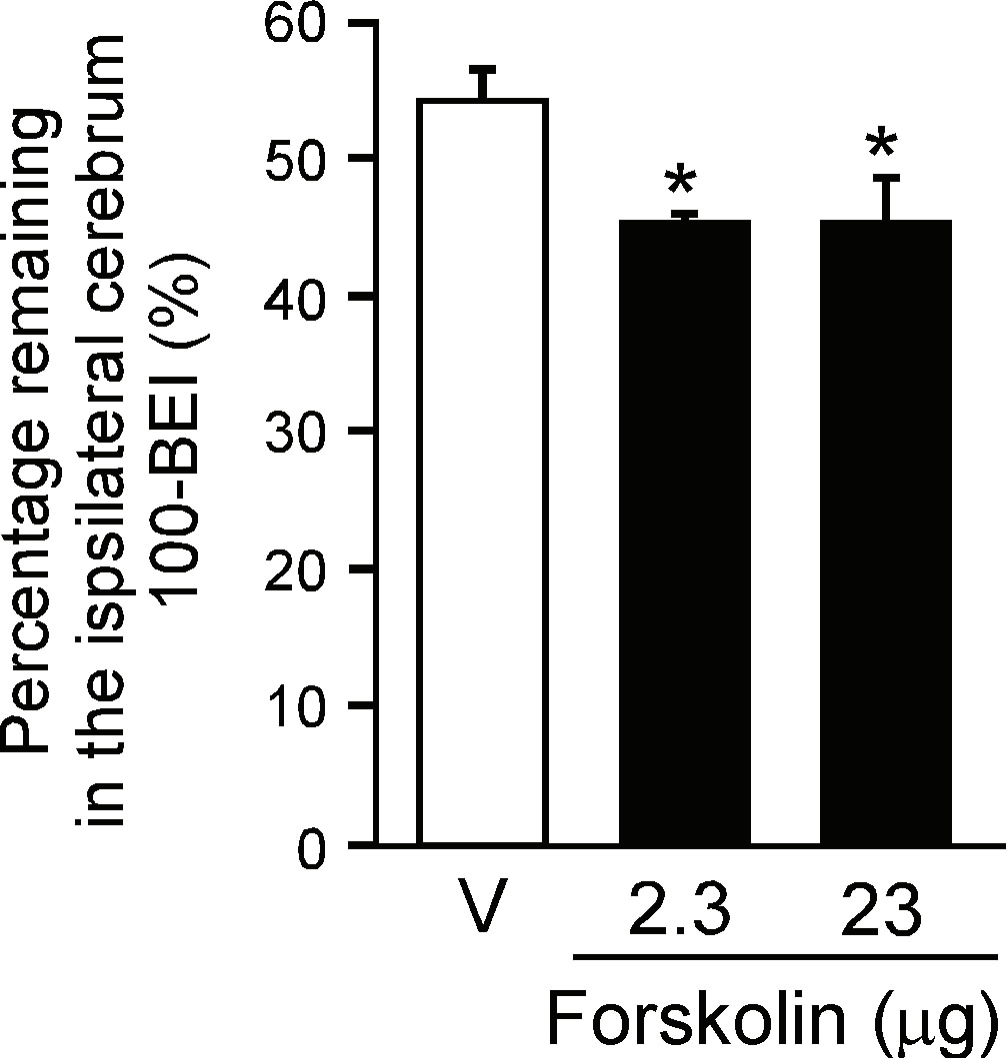
**Effect of forskolin on amount of [^125^I]hAβ(1-40) remaining in the ipsilateral cerebrum**. Mice received i.p. injection of forskolin (2.3 and 23 μg dissolved in 100 μL of H_2_O containing 25% DMSO) or vehicle (V, 100 μL of H_2_O containing 25% DMSO). After 24 h, a mixture of [^125^I]hAβ(1-40) (0.012 μCi) and [^3^H]dextran (0.12 μCi) dissolved in 0.30 μL of ECF buffer was injected into the S2 region of the brain. Data, obtained at 60 min after intracerebral microinjection, are presented as the mean ± SEM (n = 4 - 5). *p < 0.05, significantly different from vehicle-treated group.

1,25(OH)_2_D3 also exerts non-genomic biological effects via a complex signaling process, which has been reported to involve increased levels of cyclic AMP (cAMP) [30] and activation of the mitogen-activated protein kinase kinase (MEK) pathway [31,32]. Since forskolin increases the level of cAMP following activation of adenylyl cyclase *in vitro*, the effect of forskolin on the remaining percentage of [^125^I]hAβ(1-40) in the ipsilateral cerebrum was examined (Figure 5). Twenty-four h after i.p. administration of single-dose forskolin (2.3 or 23 μg/mouse), the percentage of [^125^I]hAβ(1-40) remaining in mouse brain was decreased by 16.4% in either case compared with the vehicle-treated group at 60 min after intracerebral [^125^I]hAβ(1-40) microinjection.

### Effect of MEK inhibitor on the forskolin-induced enhancement of [^125^I]hAβ(1-40) internalization by TM-BBB4 cells

**Figure 6:**
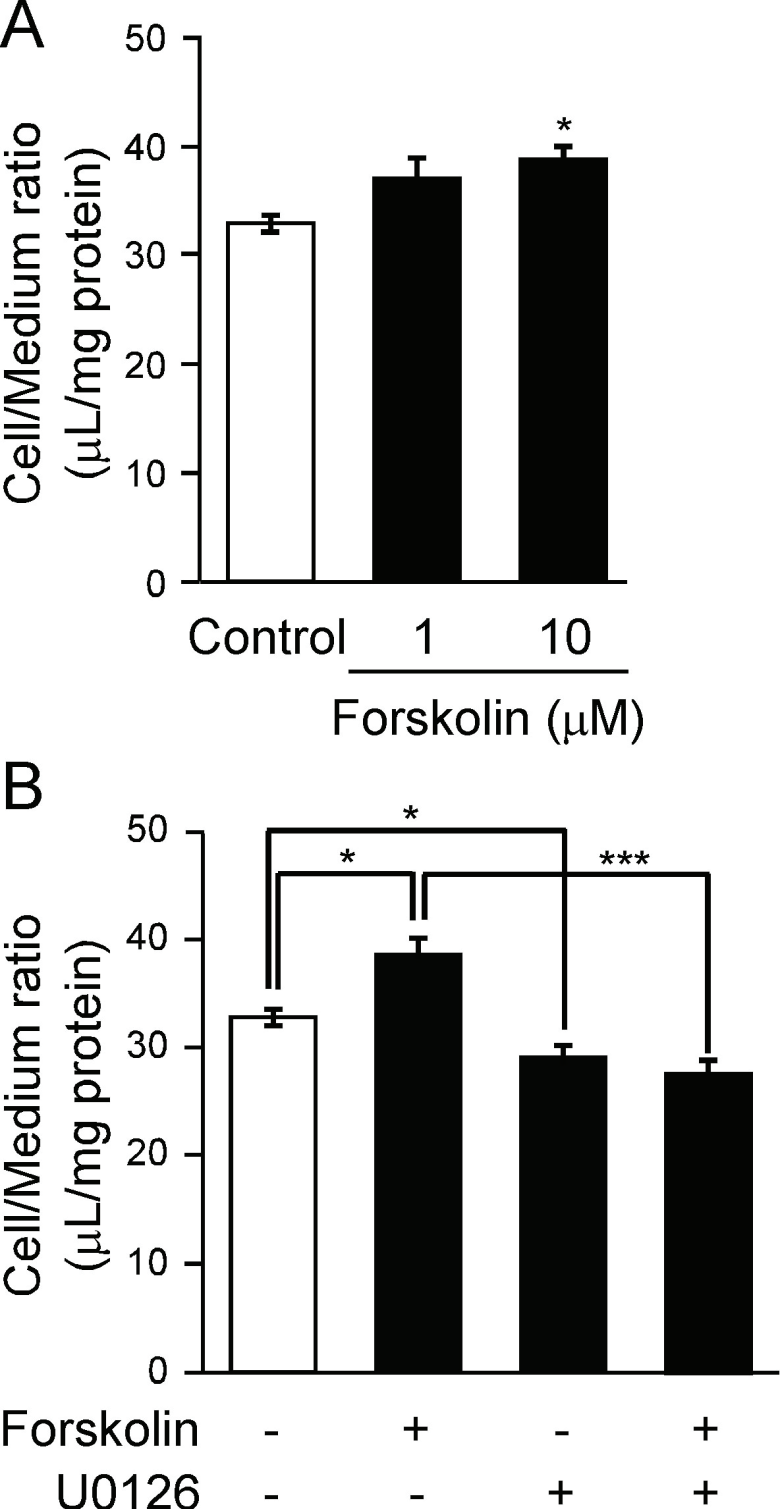
**Involvement of ERK pathway in the uptake of [^125^I]hAβ(1-40) by TM-BBB4 cells**. (A) Effect of forskolin on [^125^I]hAβ(1-40) internalization in TM-BBB4 cells. TM-BBB4 cells were treated with 1 or 10 μM forskolin for 24 h in culture medium containing 0.1% DMSO. The acid-resistant binding of [^125^I]hAβ(1-40) (0.4 nM) was measured at 3 min in TM-BBB4 cells. Each bar represents the mean ± SEM (n = 4). (B) Effect of MEK inhibitor, U0126, on [^125^I]hAβ(1-40) internalization in TM-BBB4 cells. TM-BBB4 cells were treated with or without forskolin (10 μM) and U0126 (25 μM) in culture medium containing 0.1% DMSO for 24 h. The acid-resistant binding of [^125^I]hAβ(1-40) (0.4 nM) was measured at 3 min in TM-BBB4 cells. Each bar represents the mean ± SEM (n = 4). *p < 0.05, *** p < 0.001, significant different from control or forskolin-treated group.

To determine whether MEK is involved in the forskolin-induced enhancement of [^125^I]hAβ(1-40) elimination from the brain, the effect of U0126, an inhibitor of MEK activation, on [^125^I]hAβ(1-40) internalization into TM-BBB4 cells was assessed. In the present study, [^125^I]hAβ(1-40) bound to the cell surface was washed off with acetate-barbital buffer (pH 3.0) after the uptake, and the acid-resistant binding represents the amount of internalized [^125^I]hAβ(1-40) in TM-BBB4 cells. Pre-treatment with forskolin (10 μM) significantly increased the acid-resistant binding of [^125^I]hAβ(1-40) by TM-BBB4 cells (Figure 6A), indicating that [^125^I]hAβ(1-40) internalization into TM-BBB4 cells was enhanced by forskolin treatment (Figure 6A). As shown in Figure 6B, U0126 (25 μM) suppressed the forskolin-induced enhancement of [^125^I]hAβ(1-40) acid-resistant binding. Pre-treatment with U0126 alone significantly reduced the acid-resistant binding of [^125^I]hAβ(1-40) by 11.2% compared with the control. This reduction could be due to inhibition of basal MEK activation and perhaps in part due to a toxic effect of U0126, since the protein content of the cells after the uptake was reduced by 19.8% by U0126 treatment (89.8 ± 6.0 μg protein/well and 72.0 ± 7.0 μg protein/well, respectively, mean ± SEM, n = 4). The acid-resistant binding of [^125^I]hAβ(1-40) was not significantly different between the group pre-treated with U0126 alone and that treated with forskolin and U0126. The protein content of the cells was not also affected significantly (72.0 ± 7.0 μg protein/well for U0126 only and 66.4 ± 3.9 μg protein/well for forskolin and U0126, mean ± SEM, n = 4). This result suggests that MEK activation is involved in the enhancement of Aβ(1-40) internalization by forskolin in TM-BBB4 cells.

## Discussion

Our results indicate that 1,25(OH)_2_D3 enhances cerebral clearance of Aβ(1-40) from mouse brain across the BBB. Specifically, we have obtained *in vivo *evidence that 1,25(OH)_2_D3 treatment significantly increased [^125^I]hAβ(1-40) elimination from mouse brain across the BBB (Figurs 1, 2), and resulted in a significant reduction in the level of endogenous soluble Aβ(1-40) in mouse brain (Figure 3). The enhancement of [^125^I]hAβ(1-40) elimination from the brain might be explained in terms of an increase of [^125^I]hAβ(1-40) efflux transport activity across the BBB, or a decrease of [^125^I]hAβ(1-40) binding to brain parenchymal cells, or both. However, the kinetic data shown in Figure 1 indicate that 1,25(OH)_2_D3 treatment increased the efflux transport activity, but did not affect the binding.

The present study examined the [^125^I]hAβ(1-40) elimination from the brain for 60 min, and considerable degradation of injected [^125^I]hAβ(1-40) might occur during this period. However, we showed previously that the elimination of [^125^I]hAβ(1-40) from mouse brain was inhibited to 21.2% by unlabeled hAβ(1-40) and that intact [^125^I]hAβ(1-40) was detected in plasma after its microinjection into rat brain [6,33]. Furthermore, the elimination of [^125^I]hAβ(1-40) from rat brain was not significantly inhibited by L-tyrosine [6]. These results suggest that the present BEI findings mainly reflect the elimination of intact [^125^I]hAβ(1-40) and possibly also to some extent partially-degraded [^125^I]hAβ(1-40), but not [^125^I]L-tyrosine, from the brain. It is also possible that free [^125^I] was generated by the degradation of [^125^I]hAβ(1-40) in the brain. It was reported that rat brain capillary endothelial cells possess iodide efflux activity and that the activity was affected by intracellular ATP level [34]. Therefore, we cannot rule out the possibility that the enhancement effect of 1,25(OH)_2_D3 shown in Figure 1 is partially due to facilitation of free [^125^I] elimination from the brain. Nevertheless, the reduction of endogenous Aβ(1-40) level in the brain by 1,25(OH)_2_D3 treatment, as shown in Figure 3, suggests that, at least, hAβ(1-40) elimination from the brain was enhanced by the treatment.

The biological effects of 1,25(OH)_2_D3 are mediated by a mixture of genomic and non-genomic actions. The genomic action of 1,25(OH)_2_D3 is mediated by binding of VDR-retinoid × receptor (RXR) complex at the vitamin D response elements on target genes. RT-PCR analysis confirmed the expression of VDR mRNA in mouse brain capillaries (Figure 4). We previously demonstrated the expression of RXRs in rat brain capillaries [35]. These results indicate that brain capillary endothelial cells possess VDR-mediated genomic pathway(s), which may be involved in the enhancement of Aβ efflux transport activity.

As regards non-genomic actions, 1,25(OH)_2_D3 has been proposed to play roles in the generation of intracellular second messengers and various signal-transduction cascades [36,37]. 1,25(OH)_2_D3 increased the intracellular level of cAMP in several cell lines as efficiently as did forskolin [30,38], and the MEK-mitogen-activated protein kinase (MAPK)-extracellular signal-regulated kinase (ERK) signaling pathway is known to be activated through elevation of intracellular cAMP. The present BEI study demonstrated that forskolin enhanced [^125^I]hAβ(1-40) elimination from mouse brain (Figure 4) and the *in vitro *uptake study showed that forskolin increased [^125^I]hAβ(1-40) internalization into TM-BBB4 cells, and this action was inhibited by MEK inhibitor (Figure 6B). These results suggest that the MEK-MAPK-ERK signaling pathway is involved in enhancing the brain-to-blood efflux transport of hAβ(1-40) at the BBB. Therefore, 1,25(OH)_2_D3-mediated enhancement of brain-to-blood hAβ(1-40) efflux transport is likely to involve both genomic and nongenomic actions of 1,25(OH)_2_D3. The enhancing effect of forskolin on [^125^I]hAβ(1-40) elimination (16.4%, Figure 5) is smaller than that of 1,25(OH)_2_D3 (25.4%, Figure 1A). This slight difference may be explained by a difference in the activating activity of 1,25(OH)_2_D3 and forskolin at different doses, although it is also possible that forskolin activated only a part of the signaling pathways activated by 1,25(OH)_2_D3.

TM-BBB4 cells retain the expression of various molecules and functions of the *in vivo *BBB [39]. However, the cells do not have polarity, and internalization into the cells may therefore reflect both uptake from the brain to the brain capillary endothelial cells and from the blood to the brain capillary endothelial cells. The effect of forskolin in enhancing the [^125^I]hAβ(1-40) internalization is likely to reflect enhanced hAβ(1-40) uptake from the brain to the cells, since *in vivo *forskolin treatment increased the elimination of [^125^I]hAβ(1-40) from the brain (Figure 5). A study of the effect of 1,25(OH)_2_D3 on the [^125^I]hAβ(1-40) internalization by TM-BBB4 failed to provide consistent results (data not shown). This may be due to the more complex activation pathways of 1,25(OH)_2_D3 than forskolin, as well as the non-polarized character of TM-BBB4 cells. Optimization of uptake conditions would be necessary for further studies. The molecules involved in hAβ(1-40) internalization in TM-BBB4 cells have not yet been identified. Our previous report using conditionally immortalized rat brain capillary endothelial cells (TR-BBB) showed that low-density lipoprotein receptor-related protein 1 indirectly contributes to [^125^I]hAβ(1-40) internalization and a P-glycoprotein substrate, verapamil, did not inhibit the uptake [40].

Soluble Aβ levels correlate with synaptic dysfunction in AD brain [41-43], and soluble Aβ dimers and oligomers initially generated from Aβ monomers are involved in the impairment of synaptic plasticity and memory [44,45]. Our present findings indicate that 1,25(OH)_2_D3 modulates brain-to-blood efflux transport of hAβ(1-40) at the BBB, and we therefore consider that impairment of 1,25(OH)_2_D3 signaling leads to a decrease in the brain-to-blood efflux transport activity of hAβ(1-40) at the BBB, which in turn results in an increase of Aβ levels in the brain. Clinical data suggest that low serum 25(OH)D3 is associated with increased risk of cognitive impairment in elderly people [10-12], and higher serum 25(OH)D3 levels were found to be associated with better cognitive test performance in AD patients [14]. Epidemiological studies support an association between VDR gene polymorphisms in the ligand-binding site and the development of late-onset AD [15,16]. These relations between vitamin D status and AD development seem likely to arise, at least partially, from impairment of the brain-to-blood Aβ efflux transport activity at the BBB due to attenuation of basal 1,25(OH)_2_D3-mediated signaling in brain capillary endothelial cells.

Reduction of Aβ in the CNS is considered to be a primary therapeutic target for AD. Based on our findings here, 1,25(OH)_2_D3 appears to be a candidate agent for reduction of Aβ(1-40) level in the brain through enhancement of Aβ elimination across the BBB. The enhancing effect of 1,25(OH)_2_D3 was observed at 24 h after administration, but was not observed at 48 h or 72 h (Figure 2B). This is likely to be due to rapid clearance of 1,25(OH)_2_D3 from the circulation, since the plasma concentration of 1,25(OH)_2_D3 reaches a maximum within 2 h and returns to baseline 24 h after i.p. administration [46]. The first experiment shown in Figure 1 was conducted at 24 h after the treatment, to allow sufficient time for functional expression, which involves following gene induction, protein synthesis and translocation. Several *in vivo *studies have examined the effect of vitamin D3 at 24 h after the last treatment [47-49].

To maintain the enhancing effect, repeated administration of 1,25(OH)_2_D3 would be necessary. However, an effective dose of 1,25(OH)_2_D3 might induce adverse effects such as hypercalcemia, because mice receiving over 0.1 μg of 1,25(OH)_2_D3 every other day for 2 weeks had hypercalcemia of over 12 mg/dL [50]. Indeed, mice exhibited reduced body weight and abnormal behavior from day 4 during treatment with 1,25(OH)_2_D3 (1 μg/day, data not shown).

Several vitamin D analogues appear to induce less hypercalcemia than 1,25(OH)_2_D3, but might retain the Aβ-transport-enhancing activity of 1,25(OH)_2_D3. However, paricalcitol, a vitamin D analogue, did not significantly enhance [^125^I]hAβ(1-40) elimination from mouse brain (data not shown). Therefore, 1,25(OH)_2_D3 and its analogues are unlikely to be candidate disease-modifying agents for AD. Nevertheless, since serum 25(OH)D3 levels tend to decrease with aging [51], maintaining a normal serum level (32 to 70 ng/mL) could be helpful for prevention of AD. Furthermore, since complex genomic and nongenomic pathways appear to be involved in the action of 1,25(OH)_2_D3, separation of these effects by structural modification might be feasible. The present *in vivo *and *in vitro *results suggest that the increase in cAMP levels by forskolin might be a candidate pathway for disease-modification of AD, although the *in vivo *effects of forskolin are still poorly understood.

## Conclusions

Our results indicate that 1,25(OH)_2_D3 plays a role in enhancing the Aβ(1-40) efflux transport process at the BBB. Compounds that activate cAMP signaling may be candidate therapeutic agents for prevention and treatment of AD through functional modulation of Aβ efflux at the BBB. TM-BBB4 cells should be a good model for studying the mechanism of hAβ(1-40) efflux transport at the BBB, and also for screening compounds that enhance the hAβ(1-40) efflux transport activity.

## List of abbreviations

AD: Alzheimer's disease; Aβ: amyloid-β peptide; BBB: blood-brain barrier; BEI: Brain Efflux Index, BEI; cAMP: cyclic AMP; DEA: diethylamine; 1,25(OH)_2_D3: 1α,25-dihydroxyvitamin D_3_; k_el_: elimination rate constant; ECF: extracellular fluid; 25(OH)D3: 25-hydroxyvitamin D_3_; hAβ(1-40): human Aβ(1-40); ERK: extracellular signal-regulated kinase; MAPK: mitogen-activated protein kinase; MEK: mitogen-activated protein kinase kinase; RXR: retinoid × receptor; S2: secondary somatosensory cortex 2; VDR: vitamin D receptor.

## Competing interests

The authors declare that they have no competing interests.

## Authors' contributions

SI and YN carried out the *in vivo *animal studies, YK and SM carried out the *in vitro *study using cells, SI participated in the study design and manuscript preparation, SO and TT supervised the study design, manuscript preparation and provision of funding. All authors read and approved the final manuscript.
